# Comparison of the effect of pressure control and volume control ventilation on endotracheal tube cuff pressure in patients undergoing general anesthesia and mechanical ventilation: a parallel randomized clinical trial

**DOI:** 10.1186/s12871-023-02263-1

**Published:** 2023-09-05

**Authors:** Shahram Nasrolahzadeh, Javad Nourian, Ahmad Khosravi, Saeed Ghasempour, Ali Abbasi, Hossein Ebrahimi

**Affiliations:** 1 Imam Hossein Hospital, Shahroud University of Medical Sciences, Shahroud, Iran; 2 Clinical Research Development Unit, Imam Hossein Hospital, Shahroud University of Medical Sciences, Shahroud, Iran; 3 Department of Epidemiology, Center for Health Related Social and Behavioral Sciences Research, Shahroud University of Medical Sciences, Shahroud, Iran; 4 Student Research Committee, School of Nursing and Midwifery, Shahroud University of Medical Sciences, Shahroud, Iran; 5 Department of Nursing, School of Nursing and Midwifery, Shahroud University of Medical Sciences, Shahroud, Iran; 6 Center for Health Related Social and Behavioral Sciences Research, Shahroud University of Medical Sciences, Shahroud, Iran

**Keywords:** Volume control ventilation, Pressure control ventilation, Endotracheal tube cuff pressure, Mechanical ventilation

## Abstract

**Background:**

Endotracheal intubation and mechanical ventilation are prevalent interventions in the operating room and intensive care unit. Recently, the complications of endotracheal tube cuff pressure have been a topic of interest. Therefore, this study compared the effect of pressure control and volume control ventilation modes on the endotracheal cuff pressure rate in patients undergoing general anesthesia and mechanical ventilation.

**Methods:**

In this triple-blinded randomized clinical trial, 50 patients undergoing open limb surgery and inguinal hernia were allocated to two groups of 25 based on inclusion criteria. After intubation, one group underwent ventilation on the pressure control ventilation mode, and the other underwent ventilation on the volume control ventilation mode. In both groups, using a manometer, the cuff’s pressure was first adjusted in the range of 25–30 cm of water. Then, the cuff pressure was measured at 10, 20, and 30 min intervals. The data were statistically analyzed using independent t-test, and two-way repeated measures ANOVA.

**Results:**

The present study’s findings showed that cuff pressure has significantly decreased over time in both study groups (*F =* 117.7, *P <* 0.001). However, a repeated measures ANOVA with a Greenhouse-Geisser correction showed no interaction between time and groups (*F =* 0.019, *P =* 0.98). The two groups had no significant difference in cuff pressure (*F =* 0.56, *P =* 0.458).

**Conclusion:**

Since the cuff pressure has been significantly reduced in both groups over time, continuous monitoring of endotracheal tube cuff pressure in patients undergoing mechanical ventilation is essential. Therefore, it is suggested to keep the cuff pressure within the recommended range to prevent complications resulting from cuff pressure reduction, such as aspiration and ventilation decrease.

**Trial registration:**

The study was registered in the Iranian Registry of Clinical Trial on 23/02/2019 (trial registration number: IRCT20181018041376N1).

## Introduction

Many patients are admitted to intensive care units, and almost all surgeries requiring general anesthesia need respiratory support by mechanical ventilation, which may cause ventilator-induced lung injury by various mechanisms. A proper artificial airway should be provided for this device to have adequate ventilation, often supplied by the endotracheal tube [1, 2]. Endotracheal intubation is a common intervention in the operating room and intensive care unit.

Recently, much attention has been paid to the complications of endotracheal cuff pressure (ETCP), especially the need to monitor ETCP in the operating room during surgery. Immediate side effects on the endotracheal and larynx were reported 15–94% after intubation. Voice violence and sore throat are 15% and 80%, respectively [3]. An excessive increase in ETCP leads to hypoperfusion, which is associated with tracheal ischemia, stenosis, necrosis, inflammation, wound, nerve damage, or fistula [3]. Conversely, if ECTP is too low, secretions can be microaspirated, leading to ventilator-associated pneumonia (VAP) [4].

Many factors can affect complications and problems in the endotracheal tube for patients who use mechanical ventilation. Some of these factors are lack of sufficient airway moisture, high content of inhalation oxygen, inadequate heat of administered gases, improper values, and pressure changes of the endotracheal tube cuff. Insufficient pressure of the endotracheal cuff causes damage to the endotracheal wall (at higher than standard cuff pressure). It also causes ventilation air leakage and the risk of aspiration of gastric contents and pneumonia caused by mechanical ventilation (at lower than normal cuff pressure) subsequently [5]. In this regard, Lizy et al. (2014) noted that the proper pressure for the tracheal tube cuff to prevent microaspiration and tracheal damage is between 20 and 30 cmH_2_O [6].

One of the affecting factors of the ETCP is positive pressure mechanical ventilation [6]. In volume control ventilation (VCV) mode, the ventilator delivers the adjusted Tidal Volume at a constant flow rate. In contrast, in pressure control ventilation (PCV) mode, the ventilator has a constant pressure over the inhalation time while the flow decreases. Compared to VCV, the gases distributed in PCV are more homogeneous in the lung, and the alveolus does not over-open. Therefore, the risk of barotrauma reduces. Both ventilation modes may be used for most patients undergoing anesthesia and routine surgery [7, 8]. Nowadays, more and better studies are needed due to widely use of ventilation modes in ventilators and the significant increase in patients’ need for mechanical ventilation. Therefore, this study compared the effect of pressure control and volume control ventilation modes on the endotracheal cuff pressure rate in patients undergoing general anesthesia and mechanical ventilation. We hypothesized that ETCP might be lower in pressure control ventilation than volume control ventilation in these patients.

## Methods

### Purpose

This study determined and compared the effect of pressure control and volume control ventilation modes on the pressure rate inside the endotracheal cuff in patients undergoing general anesthesia and mechanical ventilation.

### Design

This parallel randomized clinical trial with the registration number IRCT20181018041376N1 was registered in the Iranian Registry of Clinical Trial on 23/02/2019. It was conducted on patients undergoing open limb surgery and inguinal hernias referred to Imam Hussein Hospital relevant to Shahroud University of Medical Sciences from April 2019 to February 2020.

### Participants

Fifty patients were selected from the study population in two groups (intervention and control) based on the desired characteristics. Inclusion criteria in this study included: 18–40 years old Patients who were ready for open limb or inguinal hernia surgery in the supine position, ASA 1 & 2 (patients of class 1 & 2 of anesthesia surgery assortment), and BMI = 18–24 [9]. Also, exclusion criteria included: patients who smoked and used narcotics, suffered from chronic obstructive pulmonary disease (COPD), and any complications during anesthesia and surgery.

### Intervention

This study was conducted on 50 patients undergoing open limb surgery or inguinal hernia who met the inclusion criteria. After obtaining verbal and written informed consent from the patient, participants were randomly assigned to intervention and control groups based on quadrigeminal blocks. The patients were intubated and connected to the ventilator in the operating room. In the intervention group, pressure control ventilation was used; in the control group, volume control ventilation was used for mechanical ventilation of patients during anesthesia.

Pre-intervention patient preparation: Both groups received the same general anesthesia. Pre- anesthesia drug was Fentanyl (2 µg/kg), to start the anesthesia was Propofol (1.5 mg/kg), muscle relaxant was Atracurium (0.5 mg/kg), The continuance of anesthetic was propofol-remifentanil and Ventilation, was with an equal mixture of oxygen and air (no N_2_O) [10]. Intubation was performed in all cases by an anesthesiologist.

Initially, the German VBM Aneroid Manometer (with a measuring range of 0-120 cm) adjusted the cuff pressure for both groups in the standard range (25 to 30 cm). The endotracheal tube was then fixed on the right side of the mouth of the patients using a band on the number 21 for 7-7.5 tubes and 23 on the 8-8.5 tubes. The correct location of the endotracheal tube was confirmed by pulmonary auscultation. The temperature and blood pressure of the patient and the percentage of received oxygen were recorded at the research’s beginning.

Any complication of intubation, anesthesia, patient ventilation, cardiovascular system, and routine surgical procedure caused the patient to be excluded from the study.

Intervention: In group P (intervention), PCV was used for mechanical ventilation during anesthesia after the patient was connected to the ventilator. This mode was activated on the ventilator, and airway pressure was adjusted to 7 ml/kg to reach the current volume.

Control: In group V (control) routine system was used for the mechanical ventilation of patients during the anesthesia. VCV, which is routine ventilation, was activated on the device for this group of patients, and the current volume was adjusted to 7 ml/kg on the ventilator.

In both study groups, respiratory rate was selected ten times per minute on the ventilator. In both groups, endotracheal tube cuff pressure was measured 10, 20, and 30 min after mechanical ventilation.

### Sample size

According to the Tyagi et al. (2011) study and the mean score of peak airway pressures in the VC and PC groups, type 1 error was 0.05, and power was 80%; the sample size in each group was determined to be 20 [11]. However, considering the possible attrition of 20%, the sample size was increased to 25 patients in each group (*N =* 50).

Alpha = 0.05 Power = 0.8 Delta=-3.1.

M_1_ = 18.7 M_2_ = 15.6

SD_1_ = 3.6 SD_2_ = 3.2

N_1_ = 20 N_2_ = 20

### Blinding

In this study, participants, the assessor of the study variables, and the analyst were blinded.

**Fig. 1:**
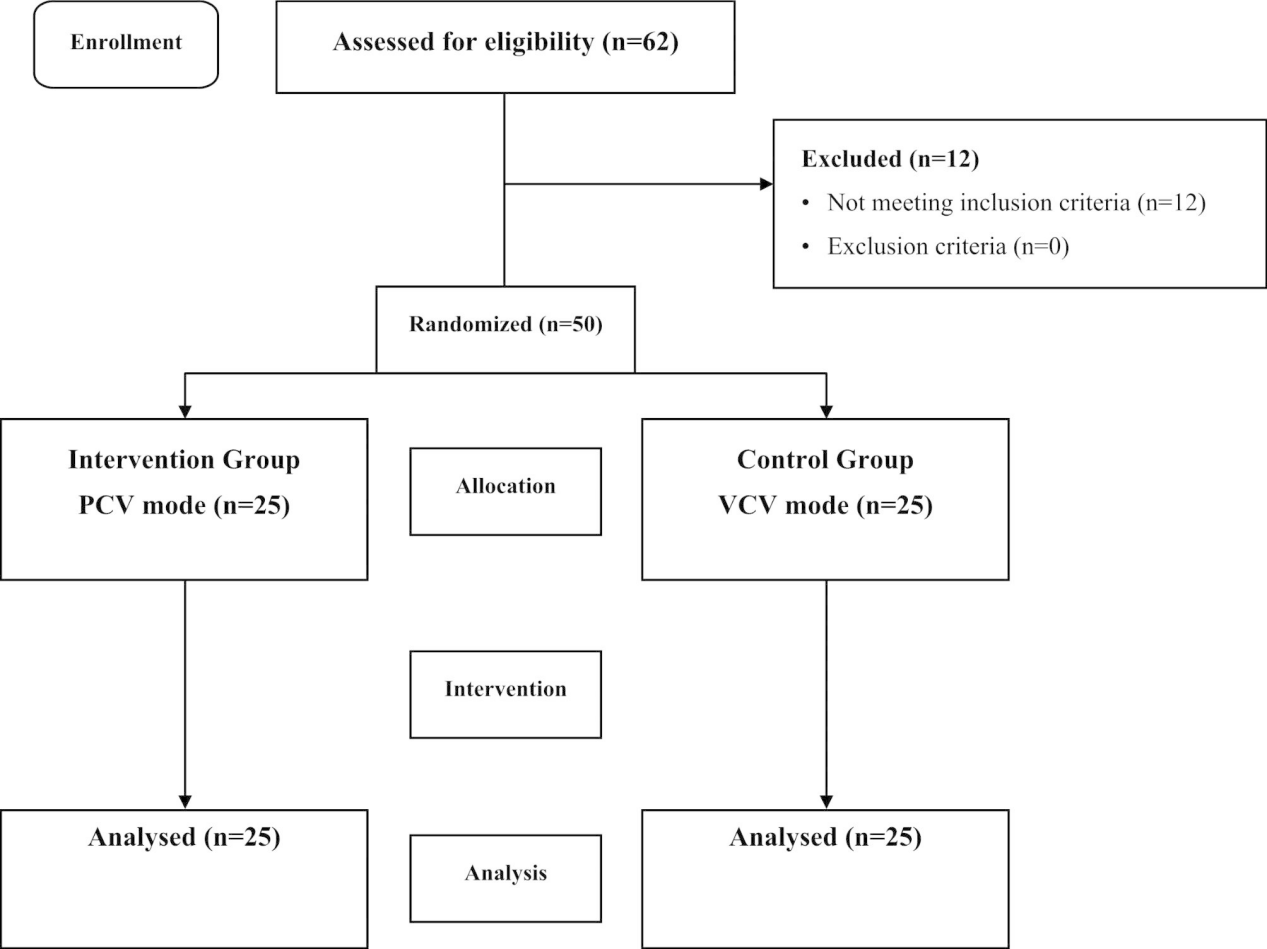
CONSORT flow diagram

Patients were selected according to the inclusion and exclusion criteria and randomly allocated to two groups of p and v using the randomly permuted blocks method. For this method, a table of 50 sequences of groups A and B was presented by consulting with a statistical consultant and using software (block number 13 and block size of 4). To ensure the concealment, each letter was placed in a sealed envelope. After closing each envelope, they were numbered based on the initial table. After selecting the eligible patient for the study, the envelope corresponding to the patient was opened, and based on the letters inside, the patient was assigned to one of two groups. The statistical consultant determined random sequence allocation. The first researcher enrolled the participants. According to the letters in each envelope written based on the specified sequence, participants were assigned to the intervention by an anesthesiologist. Data were collected and recorded by a trained individual blinded to the type of intervention performed during anesthesia (Fig. 1).

### Ethical consideration

This study was approved under the Code of Ethics IR.SHMU.REC.1397.174 at the Ethics Council for Biomedical Research at Shahroud University of Medical Sciences.

### Statistical methods

The data were analyzed by descriptive statistics (frequency, percentage, mean, and standard deviation). Inferential statistics such as independent t-test (to compare quantitative variable between two groups), and repeated measure ANOVA (for the correlated data) using SPSS software version 22. The significance level of the tests was considered to be 0.05.

## Results

The study participants included 50 patients undergoing open limb surgery and inguinal hernia. The mean age of the patients under study was 34.84 ± 6.626 years in the test group and 32 ± 8.411 years in the control group, and most of the participants were male (60%). The evaluation of demographic characteristics, such as age, sex, and BMI, showed no significant difference between the two groups in terms of demographic variables (Table 1).

**Table 1:** Comparison of demographic characteristics of study participants in two groups

Group	Pressure control ventilation		Volume control ventilation		Independent t-test
Demographic Characteristics	Mean	SD	Mean	SD	
Age	32	8.41	34.84	6.62	**t** = 1.32 **P** = 0.19
Height	172.40	7.71	170.28	8.89	**t**=-0.90 **P** = 0.37
Weight	66.40	7.75	67.84	7.60	**t** = 0.66 **P** = 0.51
BMI	22.31	1.79	23.34	0.94	**t** = 1.53 **P** = 0.05
Heart rate	88.68	9.92	87.68	14.65	**t**=-0.28 **P** = 0.77
Systolic blood pressure	125.36	13.06	124.76	18.24	**t**=-0.13 **P** = 0.89
Diastolic blood pressure	77.32	9.87	77.60	10.28	**t** = 0.09 **P** = 0.92
Endotracheal tube size	7.86	0.22	7.80	0.25	**t**=-0.88 **P** = 0.38
Tidal volume	492.84	76.57	523.8	56.56	**t** = 1.53 **P** = 0.13
Arterial Saturation Percentage	98.76	0.77	98.60	0.86	**t**=-0.67 **P** = 0.49

BMI: Body Mass Index; SD: Standard Deviation; P: p-value

**Fig. 2:**
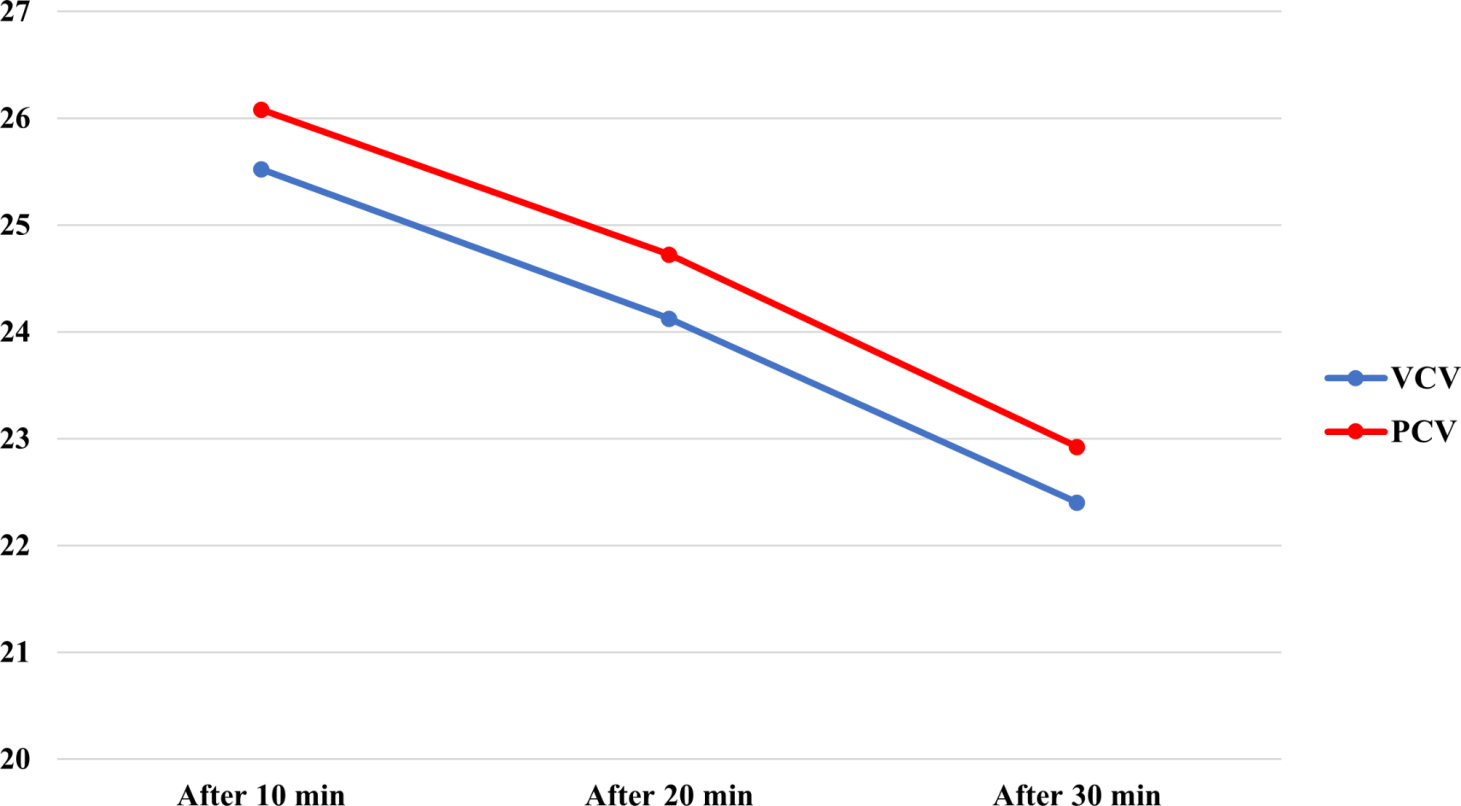
Comparison of cuff pressure 10, 20, and 30 min after ventilation in two groups

In the present study, after intubation, the mean cuff pressure at zero time was 27.16 cmH_2_O in the volume control group and 27.64 cmH_2_O in the pressure control group. Then, at 10, 20, and 30 min later, when the cuff pressure was measured again in two groups, it was respectively 25.52, 24.12, and 22.40 in the volume control group, and in the pressure control group, it was 26.08, 24.72, and 22.92, respectively. The results of the present study showed that at 30 min after ventilation, both groups had the lowest cuff pressure (Fig. 2). Using repeated measures, ANOVA showed no significant interaction between time and group (*F =* 0.019, *df =* 1.63, *P =* 0.98). A Greenhouse-Geisser correction showed that mean cuff pressure did not differ significantly between the two groups (*F =* 0.56, *df =* 1, *P =* 0.458). In both volume control and pressure control groups, the cuff pressure significantly decreased over time (*F =* 117.7, *df =* 1.63, *P <* 0.001) (Table 2).

**Table 2:** Comparison of mean and standard deviation of cuff pressure 10, 20, and 30 min after the onset of Ventilation mode in Volume and Pressure Control Groups (SD ± Mean)

Time	10 min	20 min	30 min
Group			
Volume Control **(n = 25)**	25.52 ± 2.56	24.12 ± 2.93	22.40 ± 2.66
Pressure Control **(n = 25)**	26.08 ± 2.39	24.72 ± 2.92	22.92 ± 3.10

n: Number; SD: Standard Deviation

## Discussion

This study showed no significant difference between the volume control and pressure control groups at 10, 20, and 30 min after the ventilation initiation. According to the literature review, it seems that there is no similar study investigating the effect of two volume and pressure modes on endotracheal tube cuff pressure, and this study is the first study to investigate the effect of two modes of volume and pressure ventilation on cuff pressure. Previous studies have indicated that the maximum airway pressure is significantly lower in the pressure control ventilation mode compared to the volume control ventilation mode. For example, a study conducted by Tyagi et al. (2011) comparing volume control and pressure control ventilation in patients undergoing laparoscopic cholecystectomy demonstrated that airway pressure was significantly lower in pressure control mode than in volume control mode [11]. On the other hand, the endotracheal tube cuff pressure is affected by airway pressure. In another study by Rosero et al. (2018) on the effects of increased airway pressure on endotracheal tube cuff pressure, it was found that it was significantly increased by increasing the maximal airway pressure [12]. In the study of Parsian et al. (2019), a significant positive relationship between airway pressure and ETCP was observed [13]. Another factor influencing ETCP is body position. In this regard, Bahonar et al. (2022) studied the effect of body position and bed head angle on intra-abdominal pressure and ETCP. The results of this study showed that the pressure of the ECTP in the lying position on the opposite side of the device is significantly higher than in other positions. Also, no significant relationship was observed between intra-abdominal pressure and tracheal tube cuff pressure [14]. Oğurlu et al. (2010) also compared volume and pressure control modes in laparoscopic pelvic surgery patients. Their results showed that airway pressure was significantly higher in the volume control group than in the pressure control group [15]. However, this study showed no significant difference in cuff pressure between the volume control and pressure control groups. This insignificance of the difference in cuff pressure between the two groups in this study may be due to the relatively short measurement time of the cuff pressure (cuff pressure in both groups was measured 10, 20, and 30 min after the mechanical ventilation initiation). Also, in this research, patients with normal lung function were enrolled. Therefore, their lung compliance and airway resistance were normal. While many ICU patients, due to reduced compliance, have high pressure during inhalation in volume ventilation, which can affect the cuff pressure. Airway resistance changes also affect cuff pressure [16]. Therefore, further studies on various patients that include the effect of lung compliance and airway resistance are required.

Based on the current study results, the passage of time significantly reduced cuff pressure in both volume control and pressure control groups. So, both groups had the lowest cuff pressure at 30 min after ventilation.

According to the results of the study by Sole et al. (2011), which evaluated an intervention to maintain cuff pressure in patients undergoing mechanical ventilation, the passage of time reduced cuff pressure significantly [4]. In their study, Saxena et al. (2022) investigated changes in ETCP during laparoscopic bariatric surgery. The results of this study showed that the ETCP varies significantly during this surgery. So that this pressure was significantly reduced during the removal of gastric calibration tubes (GCTs) and the release of the carbo peritoneum [17]. Also, the results of the study by Athiraman et al. (2015), in which they examined cuff pressure changes with position change in patients undergoing neurologic surgery, showed that cuff pressure decreased significantly over time [18]. The results of the current study are in line with the results of these studies. Several factors affect reducing ETCP over time. For example, the endotracheal tube material can effectively maintain pressure. Also, previous studies have shown that each cuff pressure measurement with a manometer can decrease the cuff pressure, and the greater number of cuff pressure measurements causes the cuff pressure to decrease further. Nseir’s study (2009) explains the relationship between the length of intubation time and the pressure drop of the endotracheal tube cuff so that the high-volume cuffs become porous with low pressure over time. Lack of reception of sedation drugs in undergoing intubation patients, coughing and lack of coordination with the ventilator also increases the airway pressure, and this discharge the air of the cuff and reduce its pressure over time [19].

## Conclusion

The results of this study demonstrate the importance of continued control of endotracheal tube cuff pressure in patients undergoing mechanical ventilation. Therefore, it is suggested to maintain the cuff pressure within the recommended range to prevent complications resulting from cuff pressure reduction, such as aspiration and ventilation decrease.

### Research limitations

In this study, patients undergoing mechanical ventilation in ICU were initially considered study patients. Still, since ICU patients are generally elderly patients with numerous underlying problems that may affect the results of the study, young patients undergoing mechanical ventilation in the operating room who were classified under ASA I and II anesthesia were used for sampling to obtain the most negligible effect (other factors affecting cuff pressure) and remove these limitations in the study results.

## Data Availability

The datasets used and/or analyzed during the current study are available from the corresponding author upon reasonable request.

## Abbreviations

ANOVA: Analysis of Variance
ETCP: Endotracheal Cuff Pressure
VAP: Ventilator-associated Pneumonia
VCV Mode: Volume Control Ventilation Mode
PCV Mode: Pressure Control Ventilation Mode
ASA: American Society of Anesthesiologists
BMI: Body Mass Index
ICU: Intensive Care Unit
COPD: Chronic Obstructive Pulmonary Disease
GCT: Gastric Calibration Tube
